# Improving interval estimation of binomial proportions

**DOI:** 10.1098/rsta.2008.0037

**Published:** 2008-04-11

**Authors:** X.H. Zhou, C.M. Li, Z. Yang

**Affiliations:** 1 VA Puget Sound Health Care SystemSeattle, WA 98108, USA; 2 Department of Biostatistics, University of WashingtonSeattle, WA 98195, USA; 3 Pfizer Inc.New York, NY 10017, USA; 4 Shandong University27 Shanda Nanlu, Jinan, Shandong 250100, People's Republic of China

**Keywords:** binomial, diagnostic accuracy, skewness, confidence interval, Edgeworth expansion

## Abstract

In this paper, we propose one new confidence interval for the binomial proportion; our interval is based on the Edgeworth expansion of a logit transformation of the sample proportion. We provide theoretical justification for the proposed interval and also compare the finite-sample performance of the proposed interval with the three best existing intervals—the Wilson interval, the Agresti–Coull interval and the Jeffreys interval—in terms of their coverage probabilities and expected lengths. We illustrate the proposed method in two real clinical studies.

## 1. Introduction

Constructing a CI for the binomial proportion is one of the most basic problems in statistics. This problem is complicated due to the lattice nature of the binomial distribution. The standard interval for the binomial proportion is the Wald interval. However, many authors have pointed out that the standard Wald interval has poor performance (e.g. Vollset 1993; Agresti & Coull 1998; Newcombe 1998; Brown *et al*. 2001). Particularly, Brown *et al*. (2001) have shown that the standard Wald interval can have a much lower coverage probability than the nominal level even for a very large sample size.

To avoid approximation, Clopper & Pearson (1934) proposed an ‘exact’ CI for the binomial proportion (see Bickel & Doksum (1977), pp. 180–181, for detail). However, several authors have shown that the Clopper–Pearson interval has a too wide interval length (Blyth & Still 1983; Agresti & Coull 1998); to reduce the conservativeness of the Clopper–Pearson interval, Blyth & Still (1983) and Duffy & Santner (1987) proposed more complex methods for constructing exact intervals that perform better than the Clopper–Pearson intervals.

Other alternative approximate intervals have also been proposed. Wilson (1927) discussed an interval based on asymptotic normality of the sample proportion and its true standard error; this interval is equivalent to the one based on the score statistics. One nice feature of the Wilson interval is that it has the shortest expected length in large samples among a certain class of intervals (Kendall & Stuart 1967, pp. 105–117). See Agresti & Coull (1998) for a detailed discussion about this procedure.

Agresti & Coull (1998) also proposed a simple ‘adjusted Wald’ interval by adding two successes and two failures to data before using the Wald formula to derive a 95% CI for the binomial proportion. The Agresti–Coull (AC) interval has the appeal of a simple presentation and preservation of the original Wald formula. Miettinen (1985) suggested using the likelihood ratio interval for the binomial proportion. Although the likelihood ratio interval has been shown to be uniformly most accurate (UMA) under some regularity conditions for continuous data (Lehmann 1986, pp. 89–93), the UMA property of the likelihood ratio interval no longer holds when data are discrete. Rubin & Schenker (1987) and Brown *et al*. (2001) proposed an alternative interval using the Bayesian approach with the non-informative Jeffreys prior, referred to as the Jeffreys interval.

Vollset (1993) evaluated the finite-sample performance of all the CIs discussed above, except the AC and Jeffreys intervals, in an extensive numerical study, and they recommended using the Wilson interval. Agresti & Coull (1998) also conducted a simulation study to compare the finite-sample performance of the AC interval with the Wilson interval and its continuity correction version, and they recommended using either the Wilson or the AC interval. Brown *et al*. (2001) compared the finite-sample performance of the AC, Wilson and Jeffreys intervals, along with six other alternative intervals, in terms of mean absolute coverage error and average expected length; after an extensive numerical analysis, they recommended the Wilson or the Jeffreys interval for small sample sizes (*n*≤40) and the AC interval for large sample sizes (*n*>40). Brown *et al*. (2002) used the Edgeworth expansion to explain theoretically why the Wald interval might perform so poorly. One main reason that the Wald interval behaves so poorly is that the binomial distribution is skewed, especially when the binomial proportion is near 0 or 1.

In this paper, we propose a new CI, called the Zhou–Li (ZL) interval, based on the Edgeworth expansion of a logit transformation of the sample proportion; our interval corrects for skewness of the binomial distribution. We show that the coverage probability of the proposed interval converges to the nominal confidence level at the rate of *n*^−1/2^. We also conduct a simulation study to compare the finite-sample performance of the ZL interval with the three best existing intervals—the Wilson, AC and Jeffreys intervals. After extensive numerical analysis, we find that the ZL interval shares the same conservative nature as the AC interval; that is, its coverage probability is generally greater than the nominal level. However, the expected interval width of the ZL interval is shorter than that of the AC interval and is almost a half shorter than that of the AC interval on average when the sample size is small. We also find that the ZL interval is comparable with the Wilson and Jeffreys intervals in terms of mean absolute error and average expected length. However, the ZL interval has better coverage accuracy than the Wilson and Jeffreys intervals, particularly when the binomial proportion is near 0 or 1.

This paper is organized as follows. In §2, we propose the ZL interval and study the rate of convergence of its coverage probability. In §3 we evaluate the finite-sample performance of the proposed interval in comparison with the three best existing intervals. In §4 we contrast the proposed intervals with the existing intervals in two real clinical studies.

## 2. A new CI

We assume that the random variable *X* has a binomial (*n*, *p*) distribution. Let $\hat{p}=X/n$, the ML estimator of *p*, and $\hat{q}=1-\hat{p}$. Since a logit transformation of $\hat{p}$, $\text{log}\;(\hat{p}/\hat{q})$, is closer to a normal distribution than $\hat{p}$, we consider the following pivotal statistics:

(2.1) $$T=\sqrt{n\hat{p}\hat{q}}\left(\text{log}\left(\frac{\hat{p}}{\hat{q}}\right)-\text{log}\left(\frac{p}{q}\right)\right).$$

Since the standard normal approximation for the distribution of *T* uses only the first two moments of *T*, to get a more accurate approximation than the normal distribution of *T*, we use the Edgeworth expansion, which allows us to use the third and fourth moments of *T* (Feller 1970).

We define

(2.2) $$q_{1}(x)=\frac{1-2p}{6\sqrt{pq}}(1-x^{2}).$$

In appendix A, we show that the studentized statistic, *T*, has the same first-order Edgeworth expansion as the non-studentized sample proportion, $\sqrt{n}(\hat{p}-p)/\sqrt{pq}$, as summarized in the following theorem.

Theorem 2.1 (2.3) $$P(T\leq x)=\Phi (x)+n^{-1/2}q_{1}(x)\phi (x)+g_{n}(p,x)\phi (x){\left(npq\right)}^{-1/2}+O(n^{-1}),$$

*where q*_1_(*x*) *stands for the error due to the skewness of the binomial distribution, and g*_*n*_(*p,* *x*) *is a periodic function of period* 1 *which takes values in* [−0.5,0.5] *and represents the rounding error*.

For the exact definition of *g*_*n*_(*p*, *x*), see Bhattacharya & Rao (1976, p. 238). We could just use the Edgeworth expansion in theorem 2.1 to correct explicitly for skewness in the binomial distribution and obtain a new two-sided 100(1−*α*)% CI for *p*.

However, since Edgeworth expansions do not necessarily converge as infinite series, a finite Edgeworth expansion is generally not a monotonic function. To overcome this problem, we apply Hall's (1992*a*) idea of using a monotone transformation of *T*. This idea uses a monotone transformation to correct for the skewness term in the Edgeworth expansion of *T*, and this monotone transformation is defined by

$$g(T)=n^{-1/2}b\hat{\gamma }+T+n^{-1/2}a\hat{\gamma }T^{2}+n^{-1}(1/3){\left(a\hat{\gamma }\right)}^{2}T^{3},$$

where *a*=−1/6; *b*=1/6; and $\hat{\gamma }=(1-2\hat{p})/\sqrt{\hat{p}\hat{q}}$. Using this monotone transformation, we obtain a two-sided 100(1−*α*)% CI for log (*p*/*q*),

$$[\text{log}\;\left(\frac{\hat{p}}{\hat{q}}\right)-n^{-1/2}{\left(\hat{p}\hat{q}\right)}^{-1/2}g^{-1}(z_{1-\alpha /2}),\;\text{log}\;\left(\frac{\hat{p}}{\hat{q}}\right)-n^{-1/2}{\left(\hat{p}\hat{q}\right)}^{-1/2}g^{-1}(z_{\alpha /2})],$$

where *z*_*α*_ is the *α* quantile of the standard normal distribution, and

$$g^{-1}(T)=n^{1/2}{\left(a\hat{\gamma }\right)}^{-1}[{\left(1+3a\hat{\gamma }(n^{-1/2}T-n^{-1}b\hat{\gamma })\right)}^{1/3}-1].$$

Taking an anti-logit transformation of this interval, we obtain a two-sided 100(1−*α*)% CI for *p*,

(2.4) $$L(x)=\left[\frac{\text{exp}\left(\text{log}\left(\hat{p}/\hat{q}\right)-n^{-1/2}{\left(\hat{p}\hat{q}\right)}^{-1/2}g^{-1}(z_{1-\alpha /2})\right)}{1+\text{exp}\left(\text{log}\left(\hat{p}/\hat{q}\right)-n^{-1/2}{\left(\hat{p}\hat{q}\right)}^{-1/2}g^{-1}(z_{1-\alpha /2})\right)},\frac{\text{exp}\left(\text{log}\left(\hat{p}/\hat{q}\right)-n^{-1/2}{\left(\hat{p}\hat{q}\right)}^{-1/2}g^{-1}(z_{\alpha /2})\right)}{1+\text{exp}\left(\text{log}\;\left(\hat{p}/\hat{q}\right)-n^{-1/2}{\left(\hat{p}\hat{q}\right)}^{-1/2}g^{-1}(z_{\alpha /2})\right)}\right].$$

We refer to this new interval as the ZL interval. Note that a function with the form exp(*x*)/(1+exp(*x*)) is always between 0 and 1. Hence, the ZL interval has one advantage of guaranteeing to be between 0 and 1.

In appendix B, we show that the coverage probability of the ZL interval converges to the nominal confidence level in the asymptotic order of *n*^−1/2^.

Theorem 2.2 $$P(p\in L)=1-\alpha +O(n^{-1/2}).$$

Since the statistic *T* becomes undefined when *x*=0 or *n*, in this case we would replace *x* by *x*+0.5 and *n* by *n*+1. We have also tried to add another constant, and our numerical analysis shows that the 0.5 value gives the best results in terms of coverage accuracy.

## 3. Finite-sample performance of the new interval

In this section, we report simulation studies that compare the finite-sample performance of the ZL interval with the three existing intervals that have been recommended to use in practice—the Wilson, AC and Jeffreys intervals. For the definition of these existing intervals, see appendix C. We set the two-sided nominal coverage level to be 95% (*α*=0.05) and took the sample size, *n*, to be 10, 15, 20, 25, 30, 40, 50 and 100; we selected 10 000 values of *p* uniformly from 0.000 099 to 0.999 999, increasing by a unit of 0.0001. For each combination of *p* and *n*, we compared the performance of the four intervals using evaluation criteria that were based on the coverage probability and the expected interval length (Vollset 1993). The coverage probability of a two-sided 95% CI, L(*x*), was computed by

(3.1) $$C_{n}(p)=E(I_{\left[p\in L(x)\right]}|k,p)=\sum_{x=0}^{n}\text{bin}(x;n,p)I_{\left[p\in L(x)\right]},$$

where $I_{\left[p\in L(x)\right]}$ is 1 if *p*∈L(*x*) and 0 otherwise, and bin(x;n,p) is the binomial probability when *X*=*x*. If we denote the lower and upper endpoints of L(*x*) by lower(*x*) and upper(*x*), respectively, we can then compute its expected length by the following formula:

$$W_{n}(p)=\sum_{x=0}^{n}\left[\text{upper}(x)-\text{lower}(x)]\text{bin}\;(x;n,p\right).$$

We first compared the performance of the four intervals in terms of three averaging performance measures of *C*_*n*_(*p*) and *W*_*n*_(*p*) over the chosen values of *p*. The first two measures were the mean absolute error and the average expected length, which were defined by

$$\int_{0}^{1}\left|C_{n}(p)-0.95\right|\;\text{d}p\;\text{and}\;\int_{0}^{1}W_{n}(p)\;\text{d}p,$$

respectively, and the last one is the proportion of the chosen values of *p* for which the coverage probability of the nominal 95% interval falls below 0.93, which was defined by

$$\frac{\text{no}.\;\text{of}\;10\;000\;p'\text{s}:C_{n}(p)<0.93}{10\;000}.$$

See Agresti & Coull (1998), Agresti & Caffo (2000) and Brown *et al*. (2001) for a discussion on the use of these measures.

**Figure 1:**
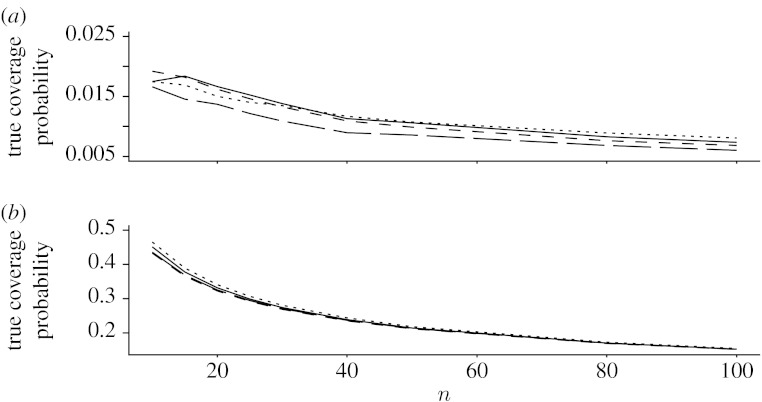
(*a*) The mean absolute errors and (*b*) average expected lengths. Solid line, ZL; dotted line, AC; dashed line, Jeffreys; long-dashed line, Wilson.

Figure 1*a* displays the mean absolute errors of the four two-sided 95% CIs for *n*=10, 15, 20, 25, 30, 40, 50 and 100. From this plot, we can see that the Wilson interval has the smallest mean absolute error, but the mean absolute errors of the four intervals are comparable in the practical sense. Figure 1*b* displays the average expected lengths of the four intervals. This plot shows that the average expected length of the ZL interval is smaller than that of the AC interval. From the plot, we also observe that the average expected length of the ZL interval is slightly larger than those of the Wilson and Jeffreys intervals, but the difference is not of practical relevance.

**Figure 2:**
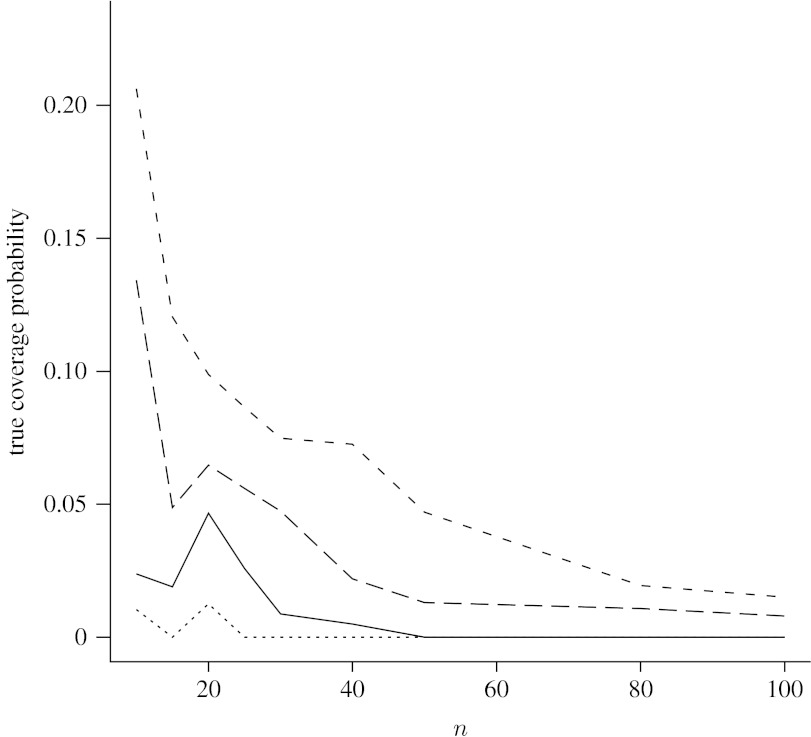
Proportions of 10 000 *p* values for which 95% nominal level intervals have actual coverage probabilities below 0.93. Solid line, ZL; dotted line, AC; dashed line, Jeffreys; long-dashed line, Wilson.

Figure 2 displays the proportions of 10 000 *p* values chosen uniformly between 0 and 1 for which the four 95% nominal level CIs have actual coverage probabilities below 0.93. From this plot, we can see that the proportion of actual coverage probabilities that are below 0.93 was small for both the AC and ZL intervals, which was less than 5%. However, the Wilson and Jeffreys intervals had much higher proportions of actual coverage probabilities that are below 0.93, especially when *n* was small. For example, when *n*=10 the proportion of actual coverage probability below 0.93 was 13.4% for the Wilson interval and 20.6% for the Jeffreys interval.

**Figure 3:**
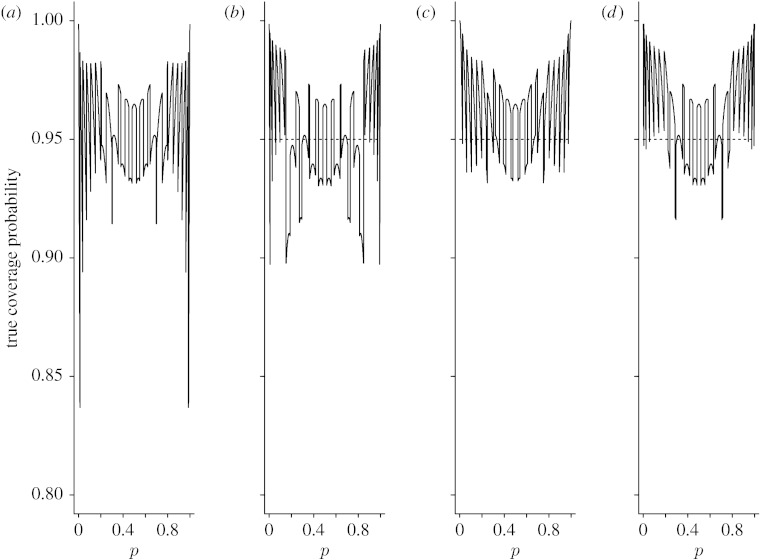
True coverage probabilities of the four two-sided 95% intervals when *n*=15. (*a*) Wilson, (*b*) Jeffreys, (*c*) AC and (*d*) ZL.

**Figure 4:**
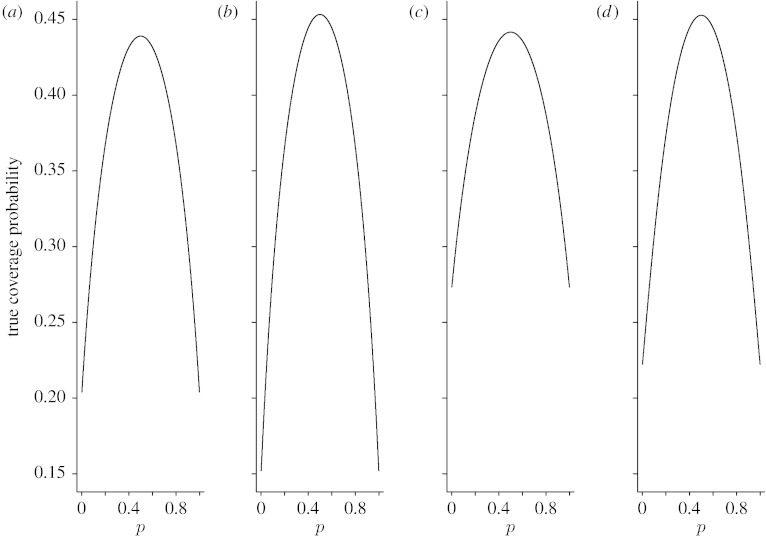
Expected widths of the four two-sided 95% intervals when *n*=15. (*a*) Wilson, (*b*) Jeffreys, (*c*) AC and (*d*) ZL.

**Figure 5:**
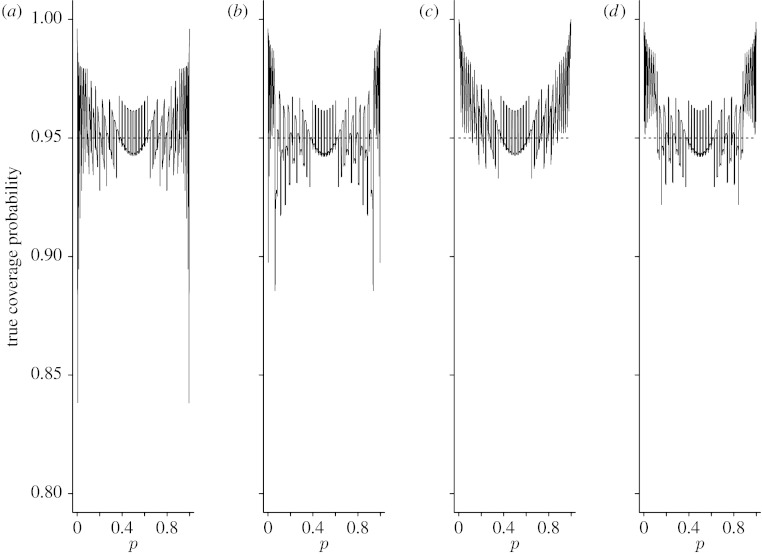
True coverage probabilities of the four two-sided 95% intervals when *n*=40. (*a*) Wilson, (*b*) Jeffreys, (*c*) AC and (*d*) ZL.

**Figure 6:**
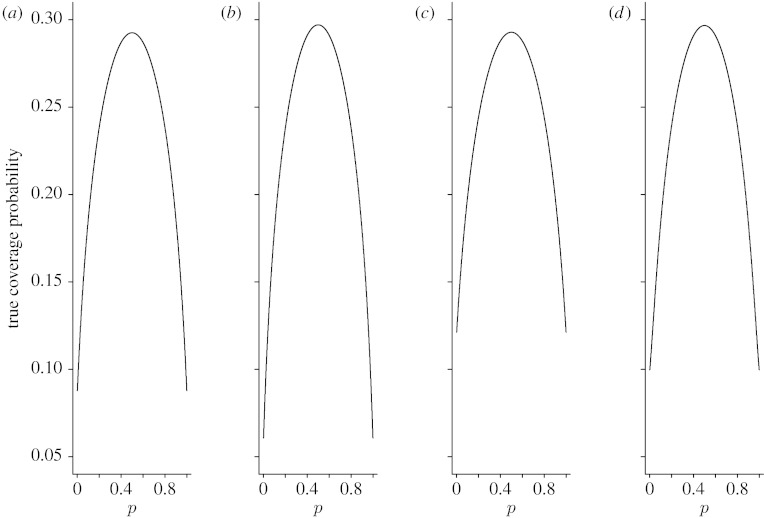
Expected widths of the four two-sided 95% intervals when *n*=40. (*a*) Wilson, (*b*) Jeffreys, (*c*) AC and (*d*) ZL.

**Figure 7:**
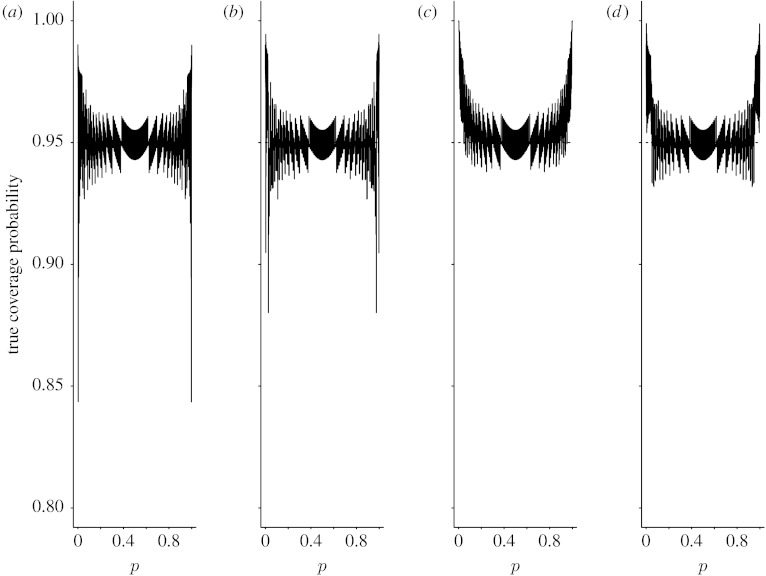
True coverage probabilities of the four two-sided 95% intervals when *n*=100. (*a*) Wilson, (*b*) Jeffreys, (*c*) AC and (*d*) ZL.

**Figure 8:**
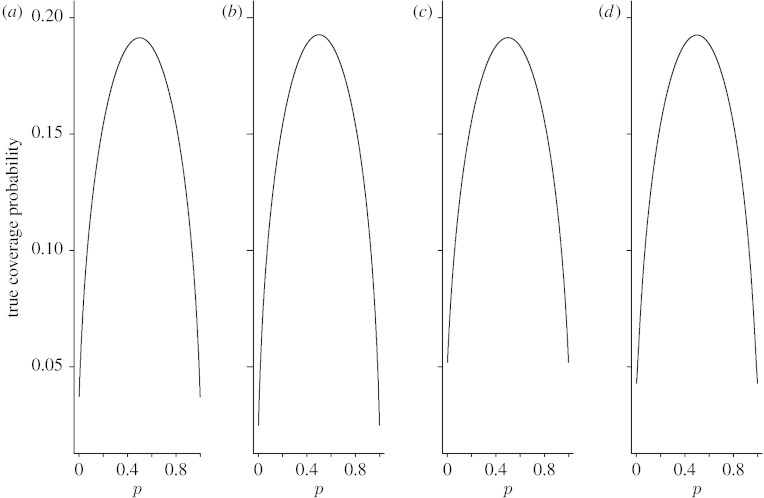
Expected widths of the four two-sided 95% intervals when *n*=100. (*a*) Wilson, (*b*) Jeffreys, (*c*) AC and (*d*) ZL.

Since averaging performance measures do not provide information on the effects of particular values of *p*, the coverage probability and expected interval length, we also plotted *C*_*n*_(*p*) and *W*_*n*_(*p*) as functions of *p* for *n*=15, 40 and 100. Figures 3–8 display the coverage probabilities and expected interval lengths of two-sided 95% CIs obtained by the four methods when *n*=15, 40 and 100.

From these figures, we can see that for most values of *p* both the Wilson and Jeffreys intervals have coverage probabilities that are below the nominal confidence level and could be significantly below the nominal confidence level when *p* is near 0 or 1, even for a sample size as large as *n*=100. Both the AC and ZL intervals have coverage probabilities that are either greater than or slightly below the nominal level. When *p* is away from 0 or 1, the coverage probabilities of both the AC and ZL intervals are very close to the nominal level; when *p* is close to 0 or 1, the coverage probabilities of the AC and ZL intervals are conservative in the sense that their coverage probabilities are greater than the nominal level.

When a CI has a conservative coverage probability, the probability that it covers the true binomial proportion is actually greater than the nominal level. However, this desirable property is usually achieved at the expense of producing a too wide CI. We saw this in the AC interval when *n* was small. For example, when *n*=15 and *p* was near 0 or 1, the expected interval length of the AC interval was much wider than those of the Wilson and Jeffreys intervals. Fortunately, for the ZL interval, its expected interval length was just slightly wider than those of the Wilson and Jeffreys intervals when *n* was small, and the difference was negligible. For large *n*, the four intervals had similar expected interval lengths.

In summary, we would make the following recommendation of the method to be used in practice. In general, without knowing the value of *p*, we would recommend the use of the Wilson interval. If we have some information about *p*, we would recommend the use of the ZL interval when *p* is close to 0 or 1 and the use of the AC interval when *p* is approximately 0.5.

## 4. Application to two real examples

We illustrate our method in two clinical studies. The first one was from a study about the effectiveness of hyperdynamic therapy in treating cerebral vasospasm (Pritz *et al*. 1996). The success of the therapy was defined as clinical improvement in terms of neurological deficits. The study reported 16 successes out of 17 patients. We were interested in deriving a two-sided 95% CI for the success rate that hyperdynamic therapy will improve neurological deficits resulting from vasospasm. Using the methods discussed in this paper, we obtained the following four 95% CIs for the success rate: (i) [0.829, 1.053] for the Wald interval, (ii) [0.730, 0.990] for the Wilson interval, (iii) [0.711, 1.009] for the AC interval, and (iv) [0.743, 0.997] for the ZL interval. It is worth noting that both the Wald and AC intervals give an upper limit that is greater than 1, the problem of overshoot. For these two intervals, we set their upper endpoints to 1.0. Because the sample proportion was close to 1, we used the ZL interval to estimate the success rate. Therefore, the 95% CI for the success rate is [0.743, 0.997]. From this interval, we conclude that the hyperdynamic therapy is a successful method to treat ischaemic neurological symptoms due to vasospasm. Although the Wilson, AC and ZL intervals all led to the same conclusion, it is worth noting that the ZL interval was completely within the AC interval.

The second study by Helmes & Fekken (1986) assessed relations between types of psychiatric disorders and the chance of receiving prescribed drugs. Among 14 psychiatric patients with affective disorder, 12 received prescribed drugs. We were interested in constructing 95% CIs for the proportion of psychiatric patients with an affective disorder who received prescribed drugs. Using the methods discussed in this paper, we obtained the various 95% CIs for *p* as follows: (i) [0.6738, 1.0404] for the Wald interval, (ii) [0.6006, 0.9599] for the Wilson interval, (iii) [0.5881, 0.9724] for the AC interval, and (iv) [0.6108, 0.9726] for the ZL interval. Once again the Wald interval gave an upper limit that is greater than 1. Although the four upper limits were similar, there were some differences among the four lower limits. For example, the lower limit of the AC interval was 4% less than that of the ZL interval and 9% less than that of the Wald interval.

## 5. Conclusions

In this paper, we proposed a ZL CI for the binomial proportion that is relatively easy to compute. Our proposed interval is based on an Edgeworth expansion of a logit transformation of $\hat{p}$. We have shown that the ZL interval converges to the nominal level at the rate of *n*^−1/2^. Based on an extensive numerical analysis of the finite-sample performance of the ZL interval and the best existing intervals, we recommend the use of the Wilson interval if there is no available information about *p*. If we have some information about *p*, we would recommend the use of the ZL interval when *p* is close to 0 or 1 and the use of the AC interval when *p* is approximately 0.5.

## Appendix A. Proof of theorem 2.1

Proof Let $T=\sqrt{n}(\text{log}\;(\hat{p}/\hat{q})-\text{log}\;(p/q))\sqrt{\hat{p}\hat{q}}$. If we let $y=\hat{p}$, we may write the statistic *T* as a function of *y*,

$$T=\sqrt{n}\left(\text{log}\;\frac{y}{1-y}-\text{log}\;\frac{p}{1-p}\right)\sqrt{y(1-y)}.$$

Writing

$$g(y)=\left(\text{log}\;\frac{y}{1-y}-\text{log}\;\frac{p}{1-p}\right)\sqrt{y(1-y)},$$

we obtain $T=\sqrt{n}g(y)$. Note that the first two derivatives of *g*(*y*) at *y*=*p* are $1/\sqrt{p(1-p)}$ and 0, respectively, and that $E{\left(y-p\right)}^{3}=pq(q-p)n^{-2}$. Therefore, expanding *g*(*y*) at *y*=*p* with a Taylor expansion, we obtain that

$$g(y)=\frac{y-p}{\sqrt{p(1-p)}}+O_{p}(n^{-2}),$$

which implies that

(A1) $$T=\sqrt{n}\frac{\hat{p}-p}{\sqrt{p(1-p)}}+O_{p}(n^{-3/2}).$$

Expression (A1) says that *T* and $\sqrt{n}(\hat{p}-p)/\sqrt{p(1-p)}$ are equivalent in probability up to *O*(*n*^−3/2^). Note that Bhattacharya & Rao (1976, p. 238) have already derived an Edgeworth expansion for $\sqrt{n}(\hat{p}-p)/\sqrt{p(1-p)}$, which has the following form:

(A2) $$P\left(\sqrt{n}\frac{\hat{p}-p}{\sqrt{p(1-p)}}\leq x\right)=\Phi (x)+n^{-1/2}q_{1}(x)\phi (x)+g_{n}(p,x)\phi (x){\left(npq\right)}^{-1/2}+O(n^{-1}),$$

where

$$q_{1}(x)=\frac{1-2p}{6\sqrt{pq}}(1-x^{2})$$

and *g*_*n*_(*p*, *x*) takes values in [−0.5,0.5] and denotes the rounding error. Therefore, applying the delta method (Hall 1992*b*, p. 34) to (A 1) and (A 2), we obtain the following Edgeworth expansion for *T*:

$$P(T\leq x)=\Phi (x)+n^{-1/2}q_{1}(x)\phi (x)+g_{n}(p,x)\phi (x){\left(npq\right)}^{-1/2}+O(n^{-1}).$$

This completes the proof of theorem 2.1. ▪

## Appendix B. Proof of theorem 2.2

To prove theorem 2.2, we first prove the following lemma.

Lemma B.1

$$P(g(T)\leq z_{\alpha })=\alpha +{\left(npq\right)}^{-1/2}g_{n}(z_{\alpha }-n^{-1/2}{\hat{q}}_{1}(z_{\alpha })+O(n^{-1}))+O(n^{-1}).$$

Proof of Lemma B.1 From theorem 2.1, we obtain that

$$P(T\leq z_{\alpha }-n^{-1/2}{\hat{q}}_{1}(z_{\alpha }))=\Phi (z_{\alpha }-n^{-1/2}{\hat{q}}_{1}(z_{\alpha }))+n^{-1/2}q_{1}(z_{\alpha }-n^{-1/2}{\hat{q}}_{1}(z_{\alpha }))\phi (z_{\alpha }-n^{-1/2}{\hat{q}}_{1}(z_{\alpha }))+n^{-1/2}{\left(pq\right)}^{-1/2}g_{n}(z_{\alpha }-n^{-1/2}{\hat{q}}_{1}(z_{\alpha }))\phi (z_{\alpha }-n^{-1/2}{\hat{q}}_{1}(z_{\alpha }))+O(n^{-1}).$$

Since both *ϕ*(*x*) and *q*_1_(*x*) are very smooth functions of *x*, using Taylor expansions, we obtain that

$$P(T\leq z_{\alpha }-n^{-1/2}{\hat{q}}_{1}(z_{\alpha }))=\alpha +{\left(npq\right)}^{-1/2}g_{n}(z_{\alpha }-n^{-1/2}{\hat{q}}_{1}(z_{\alpha }))+O(n^{-1}).$$

Since

$$P(g(T)\leq z_{\alpha })=P(T\leq g^{-1}(z_{\alpha })),$$

to show the expression in lemma B.1, we first expand

$${\left[1+3a\hat{\gamma }(n^{-1/2}x-n^{-1}b\hat{\gamma })\right]}^{1/3}-1.$$

Using a Taylor series expansion on the function (1+*y*)^1/3^, we show that

$${\left[1+3a\hat{\gamma }(n^{-1/2}x-n^{-1}b\hat{\gamma })\right]}^{1/3}-1=n^{-1/2}(a\hat{\gamma })x-n^{-1}(a\hat{\gamma })[b\hat{\gamma }-(a\hat{\gamma })x^{2}]+O_{p}(n^{-3/2}).$$

Therefore, we obtain that

$$g^{-1}(x)=x-n^{-1/2}q_{1}(x)+O(n^{-1}),$$

which implies the expression in lemma B.1. ▪

Proof of theorem 2.2 Using the result in lemma B.1, we obtain that

$$P(p\in L(x))=P(z_{\alpha /2}\leq g(T)\leq z_{1-\alpha /2})=P(T\leq z_{1-\alpha /2}-n^{-1/2}{\hat{q}}_{1}(z_{1-\alpha /2}))-P(T\leq z_{\alpha /2}-n^{-1/2}{\hat{q}}_{1}(z_{\alpha /2}))=1-\alpha +{\left(npq\right)}^{-1/2}[g_{n}(z_{1-\alpha /2}-n^{-1/2}{\hat{q}}_{1}(z_{1-\alpha /2}))-g_{n}(z_{\alpha /2}-n^{-1/2}{\hat{q}}_{1}(z_{\alpha /2}))]+O(n^{-1}).$$

Since *g*_*n*_(*x*) is a periodic function of period 1, *g*_*n*_(*x*) is bounded. Hence, we obtain $P(p\in L(x))=1-\alpha +O(n^{-1/2})$. This completes the proof. ▪

## Appendix C. Existing CIs

The Wald interval can be derived by inverting the *Z*-score test with the estimated standard error, and its 100(1−*α*)% two-sided CI has the following simple form:

$$\hat{p}\pm z_{1-\alpha /2}n^{-1/2}\sqrt{\hat{p}(1-\hat{p})}.$$

The 100(1−*α*)% two-sided Wilson interval for *p* has the following form:

$$\frac{\hat{p}+(z_{1-\alpha /2}^{2}/2n)\pm z_{1-\alpha /2}n^{-1/2}\sqrt{\hat{p}(1-\hat{p})+(z_{1-\alpha /2}^{2}/4n)}}{1+(z_{1-\alpha /2}^{2}/n)}.$$

The two-sided 100(1−*α*)% Jeffreys interval has the lower and upper endpoints as *L*_J_(*X*) and *U*_J_(*X*), respectively. Here, $L_{\text{J}}(X)=B(\alpha /2;X+1/2,n-X+1/2)$ if *X*≠0 and 0 otherwise, and $U_{\text{J}}(X)=B(1-\alpha /2;X+1/2,n-X+1/2)$ if *X*≠*n* and 1 otherwise, respectively, where *B*(*α*; *m*_1_, *m*_2_) denotes the *α* quantile of a *Beta*(*m*_1_, *m*_2_) distribution.
